# Osteomyelitis or Charcot neuro-osteoarthropathy? Differentiating these disorders in diabetic patients with a foot problem

**DOI:** 10.3402/dfa.v4i0.21855

**Published:** 2013-11-05

**Authors:** Bulent M. Ertugrul, Benjamin A. Lipsky, Oner Savk

**Affiliations:** 1 Department of Infectious Diseases and Clinical Microbiology, School of Medicine, Adnan Menderes University, Aydin, Turkey; 2 Department of Medicine, University of Geneva, Geneva, Switzerland; 3 Division of Medical Sciences, University of Oxford, Oxford, UK; 4 Department of Orthopedics and Traumatology, School of Medicine, Adnan Menderes University, Aydin, Turkey

**Keywords:** diabetic foot, osteomyelitis, *Charcot neuro-osteoarthropathy*

## Abstract

Both osteomyelitis and Charcot neuro-osteoarthropathy (CN) are potentially limb-threatening complications of diabetic neuropathy, but they require quite different treatments. Almost all bone infections in the diabetic foot originate from an infected foot ulcer while diabetic osteoarthropathy is a non-infectious process in which peripheral neuropathy plays the critical role. Differentiating between diabetic foot osteomyelitis and CN requires careful evaluation of the patient, including the medical history, physical examination, selected laboratory findings, and imaging studies. Based on available studies, we review the approaches to the diagnostic differentiation of osteomyelitis from CN of the foot in diabetic patients.

An estimated 12%–25% of persons with diabetes suffer from foot problems, with foot ulceration being the most common and often leading to the most severe complications (1–3). At presentation for medical care, clinical evidence of infection is present in more than half of diabetic foot ulcer cases. Depending on the severity of infection, between 20% and 60% of these foot infections are accompanied by underlying bone infection (4–6). Osteomyelitis almost always occurs as a consequence of contiguous spread of infection from soft tissue to bone; its presence increases morbidity, the likelihood of requiring lower extremity amputation and consequently patient mortality (7, 8). Diabetic foot osteomyelitis (DFO) is invariably treated with antibiotic therapy, usually in conjunction with surgical debridement or resection.

Charcot neuro-osteoarthropathy (CN) is an infrequent but severe complication of diabetic peripheral neuropathy that is estimated to affect 0.8%–8% of the diabetic population (9). A higher percentage of cases are found when advanced imaging studies are used for diagnosing foot problems, and the incidence of CN appears to be increasing (9, 10). In the acute stage, CN is treated by immobilisation and pressure off-loading, often combined with various bone-enhancing medications.

In diabetic patients seen for foot complications it is often difficult, especially at initial presentation, to differentiate DFO and CN. As the approach to treatment is quite different, and largely determines the outcome, it is important for clinicians to know how to diagnose each of these entities.

## Differential diagnosis

### History and physical examination

DFO is almost always caused by bacteria, either with a single organism or as part of a polymicrobial infection. While bacteria may involve bone by the haematogenous route, almost all cases occur by spread of infection from adjacent soft tissue. Thus, a history, or the presence, of a local ulceration (usually of a toe or metatarsophalangeal joint, but occasionally the calcaneus) or a ‘sausage toe’ appearance (swollen, erythematous digit lacking normal contours) is a characteristic of DFO (11, 12). A history of a previous ipsilateral ulcer or amputation or an ulcer of long duration or overlying bone is suggestive of DFO (8, 13). Patients with DFO are usually not febrile and may lack local signs of inflammation in the wound (14). Newman et al. reported that an ulcer size >2 cm^2^ had a diagnostic sensitivity for DFO of 56% and specificity of 92% (15). Similarly, we found these rates were 88% and 77%, respectively, and also demonstrated that the mean ulcer size was 6.21 cm^2^ in patients with DFO but only 1.81 cm^2^ in patients without DFO (13). A recent, multi-center study found that ulcer size >4.5 cm^2^ increased the risk of DFO threefold (8). DFO is also more likely to be present when the foot ulcer is deeper than 3 mm, compared with a shallower ulcer (82% vs. 33%, respectively) (15). Thus, cut-off values of >2 cm^2^ for the ulcer size and >3 mm for the ulcer depth are useful in diagnosing DFO (1, 5, 11, 14, 16–22).

Another useful clinical finding is whether or not bone is palpable (perceived as a hard, gritty surface) on inserting a blunt metal probe into a diabetic foot wound, called the ‘probe-to-bone’ (PTB) test. The original report of this test by Grayson et al. (23) suggested it had a high positive predictive value, but in subsequent studies the diagnostic sensitivity has ranged from 38% to 94% and the specificity from 85% to 98% (Table 1) (21, 23–29). While some still have doubts about the diagnostic value of PTB (30), it appears to be highly dependent on doing the test properly (especially using a metal probe) and on the pre-test probability (prevalence in the tested population) of DFO. In a population in which DFO is frequent, a positive test markedly increases the likelihood that DFO is present, while a negative test largely excludes DFO when the pre-test probability is low. A meta-analysis supported the value of the PTB test, as well as the other physical examination findings mentioned above (31). The value of the PTB is more plausible because it is reasonable to assume that if the probe can reach the bone, so can infectious bacteria (32).

**Table 1:** Performance characteristics of probing to bone for the diagnosis of osteomyelitis

Reference (first author, reference number)	Number of patients (ulcer type)	Sensitivity (%)	Specificity (%)	PPV (%)	NPV (%)	Prevalence (%)
Grayson et al. (23)	76 (I)	66	85	89	56	66
Shone et al. (28)	81 (A)	38	91	53	85	24
Lavery et al. (25)	247 (A)	87	91	57	98	12
Morales Lozano et al. (26)	132 (I)	94	98	95	91	80
Aragon-Sanchez et al. (24)	327 (I)	95	93	97	83	74
Mutluoglu et al. (27)	65 (I)	66	84	87	62	60

A = all diabetic foot ulcers; I = infected ulcers only; PPV = positive predictive value; NPV = negative predictive value; prevalence = the percent of patients studied who had osteomyelitis.

Almost all cases of DFO represent chronic infection by the time it is discovered. CN, however, can present either as an acute illness or a chronic condition, although sometimes these two phases appear to overlap (10, 33). Unlike osteomyelitis, which typically involves the toes and forefoot, CN typically causes bony destruction in the midfoot (34). In acute CN the foot is warm (usually >2*°*C higher in the affected than the unaffected foot), indurated and erythematous. There is usually a history of foot trauma (35–37), but this condition may mimic other causes of acute inflammation, such as cellulitis, acute venous thrombosis, or gout. The likelihood that this acute inflammatory condition is infectious is greatly reduced by the absence of the current or recent foot ulceration (10) and by the observation that when the affected leg is kept elevated there is a decrease in erythema (38). In CN, there is always evidence of a dense peripheral neuropathy, but arterial blood flow (as measured by pedal pulses) is usually normal, or even enhanced.

**Fig. 1:**
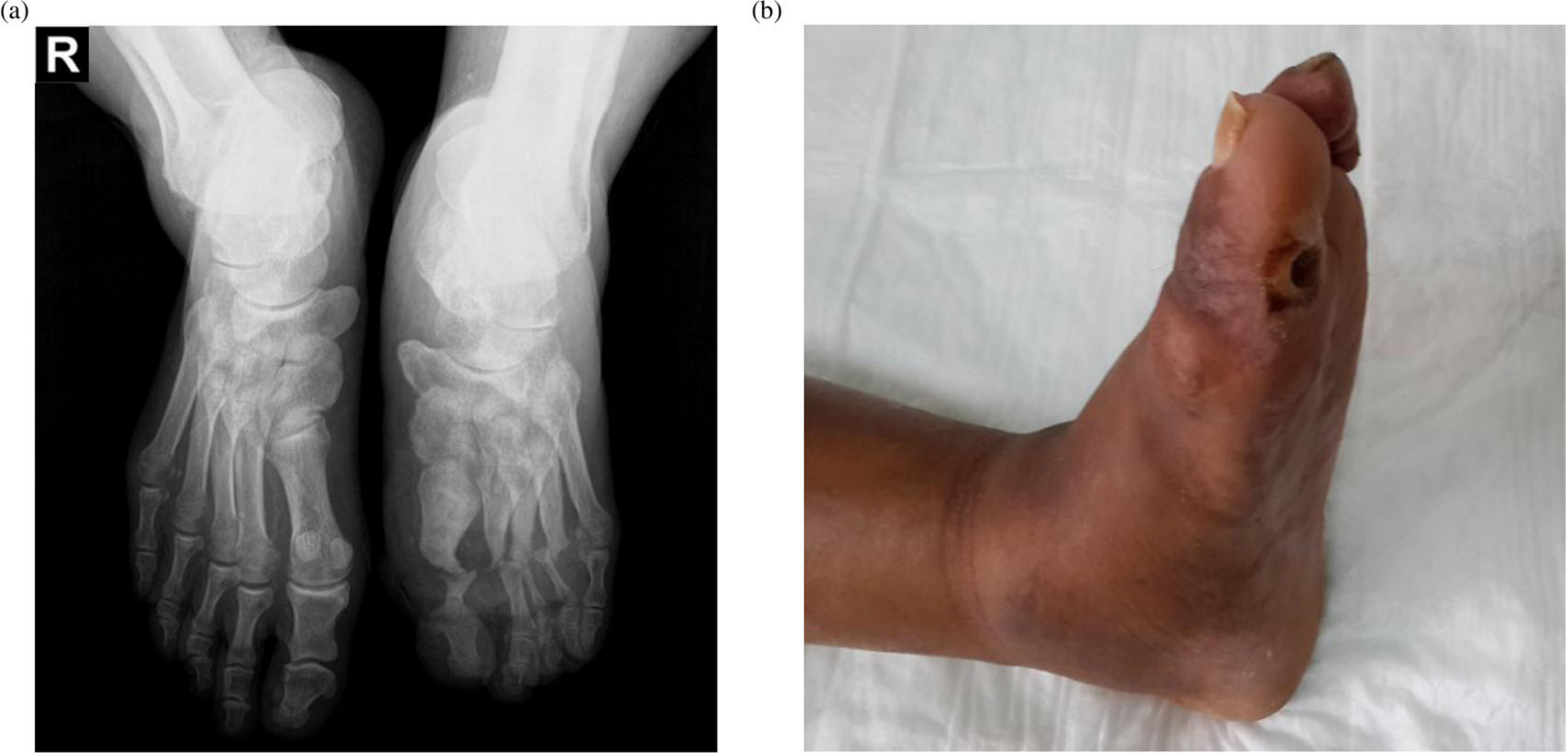
(A) Plain radiographs with the left foot showing typical bony changes in Charcot neuro-osteoarthropathy (bone destruction, joint fragmentation, and dislocation). (B) Photograph of the same patient's left foot with great toe and midfoot deformities, including collapsed arch.

In chronic CN, there is typically a diminution of the local inflammatory changes but progression of bony changes that lead to foot deformities (33). These can be slight or grossly evident, depending on the chronicity of the problem, the anatomical site involved and how appropriately the acute phase was treated (37). In most cases, the overall foot architecture is eventually deformed, especially in the midfoot; this most characteristically leads to collapse of the arch, producing a so-called rocker bottom foot (Fig. 1), (34). This in turn leads to high pressures on the midfoot soft tissues during standing and walking making the area prone to ulceration (10).

### Laboratory findings

#### Haematological tests

Certain laboratory markers may assist in differentiating DFO and CN, mostly related to the fact that the former is usually associated with elevated systemic inflammatory markers while the latter, notwithstanding the local inflammation in the acute stage, is not. A substantially elevated (>70 mm/hour) erythrocyte sedimentation rate (ESR), in the absence of another obvious explanation, supports a diagnosis of DFO (13, 15, 18, 39–43). ESR remains elevated in inadequately treated DFO, but slowly declines with appropriate therapy.

Blood leukocyte counts, serum C-reactive protein (CRP) and procalcitonin concentrations are also usually normal in CN while high in DFO, but these laboratory parameters are relatively non-specific (44). In one study of severe diabetic foot infections, both the leukocyte count and the CRP level were higher in those with exclusively soft-tissue infection than in those with concomitant osteomyelitis (45). In another study (46) leukocyte count, CRP, procalcitonin, and ESR levels were each significantly higher in patients with foot infections, including osteomyelitis, than patients without foot infection. Because DFO is usually a chronic condition, acute infection markers, such as the leukocyte count and procalcitonin, are generally within normal ranges (47, 48). Combined with clinical findings (as discussed above) a highly elevated ESR is likely to suggest osteomyelitis than cellulitis (39).

High CRP levels and elevated leukocyte counts are not common in CN (49). In one study, levels of bone-specific alkaline phosphatase (a marker of bone formation) and urinary deoxypyridinoline (a marker of bone resorption) were found to be increased in acute CN compared to diabetic persons without CN, reflecting ongoing bone turnover and remodelling (50). Two other studies found an increase in the bone resorption marker pyridinoline cross-linked carboxy-terminal telopeptide domain of type 1 collagen in acute CN (51, 52). Preliminary reports suggest, however, that conventional markers of bone turnover are of no value in differentiating osteomyelitis from Charcot arthropathy (53).

#### Bone biopsy

The criterion standard for diagnosing DFO is demonstrating positive findings on a bone specimen for both culture and histopathology. Bone culture alone has been reported to have a sensitivity of 92% and a specificity of 60%. The major advantage of bone culture is that it is the only method to define the causative pathogen(s), thereby allowing determination of their antibiotic sensitivities and accurate targeting of therapy (54, 55). Bone samples can be obtained during an open operative procedure or by percutaneous biopsy (34). Biopsy should be done through clinically uninfected skin after skin antisepsis, can be undertaken at the bedside or in the radiology suite with imaging guidance, and often does not require anaesthesia (because of sensory neuropathy). Studies have shown that cultures of overlying soft tissue or sinus tracts are not sufficiently accurate in predicting bone pathogens (56, 57). Clinicians should note that bone specimens may yield false-positive results because of contamination by wound-colonising flora, or false-negative results because of sampling errors, prior antibiotic therapy or a failure to isolate fastidious organisms (5).

Because of the potential problems noted with bone culture, many favour histopathology as the gold standard in diagnosing osteomyelitis (5, 11, 14, 16, 17, 22, 48). Characteristic histological findings include aggregates of inflammatory cells (neutrophils, lymphocytes, histiocytes, and plasma cells), erosion of trabecular bone and marrow changes, including loss of normal fat, fibrosis, and reactive bone formation (58). Although bone biopsy is safe, it does require some time, skill, and expense; it is therefore most recommended in specific circumstances, as outlined in Table 2, (11). Biopsying bone is not generally recommended in suspected CN, but reports of histological examination of surgical specimens reveal that osteoclasts significantly outnumber osteoblasts in reactive bone (50).

**Table 2:** Clinical situations in which diagnostic bone biopsy is particularly useful (11)

The patient or provider prefers a definitive diagnosis to justify the choice of early surgery rather than prolonged antibiotic treatment
Available cultures of soft tissue or blood suggest a high risk of osteomyelitis caused by an antibiotic-resistant organism
There is progressive bony deterioration or persistently elevated inflammatory markers during empiric or culture-directed therapy (consider surgical resection)
The bone suspected of being infected is a planned target for insertion of orthopaedic metalware

### Imaging studies

Plain radiology is almost always the first diagnostic test when evaluating for bone involvement in the diabetic foot. The accuracy of plain radiography for early diagnosis is only about 50%–60%, with a sensitivity of around 60% and a specificity of around 80% (59, 60). The most common findings of osteomyelitis on plain radiographs are demineralisation, periosteal reaction, and cortical destruction (14, 58), but these findings do not generally become visible until the second or third week following infection, as it requires 40%–50% loss of bone tissue to see them. Thus, for diagnosing acute osteomyelitis more advanced imaging studies are needed. Nuclear imaging studies have been used for decades and are generally widely available. Three-phase bone scintigraphy has a high sensitivity (80%–100%), but poor specificity (25%–60%) (61). Causes of false-positive results include trauma, arthritis, remodelling bone, recent surgery and CN, but negative results essentially exclude infection (62). Labelled leukocyte scintigraphy is similarly sensitive, but more specific. White blood cells can be labelled with various substances, but most often used are ^99m^Technetium or ^111^Indium. One meta-analysis reported a sensitivity for ^99m^Tc-labelled leukocyte scintigraphy of 86%, a specificity of 85%, and a positive predictive value of 90% (63). Another found that ^111^Indium-radiolabelled leukocytes scans had a pooled sensitivity of 74% and a specificity of 68% (31). ^99m^Tc labelling appears to provide superior physical characteristics, leading to better spatial resolution than ^111^In (63).

When an advanced imaging test is needed, magnetic resonance imaging (MRI) has emerged as the best currently available, for detecting both bone involvement and soft-tissue detail. In one meta-analysis, the sensitivity of MRI for diagnosing DFO was 90% while the specificity was 79% (31). Characteristic features of osteomyelitis and CN on plain X-ray and MRI are summarised in Table 3, (5, 64–66). In comparing the two imaging methods, the main advantage of MRI is its ability to identify the extent of the involved area, while labelled leukocyte scintigraphy may have better performance in differentiating osteomyelitis from CN and (unlike MR) it can be used in patients with metal implants (14, 58).

**Table 3:** Characteristic features of osteomyelitis and Charcot neuro-osteoarthropathy on plain X-ray and magnetic resonance imaging [adapted from Lipsky et al. (5), Cavanagh et al. (64) Marcus et al. (65), Tan et al. (66)]

Plain radiographs*	

Osteomyelitis	Charcot neuro-osteoarthropathy
Periosteal reaction or elevation Loss of cortex with bony erosion Focal loss of trabecular pattern or marrow radiolucency New bone formation Bone sclerosis with or without erosion Sequestrum: devitalised bone with radiodense appearance that has become separated from normal bone Involucrum: a layer of new bone growth outside existing bone resulting from the stripping off of the periosteum and new bone growing from the periosteum Cloacae: opening in involucrum or cortex through which sequestra or granulation tissue may be discharged	Non-specific changes: Periostal reaction Traumatic fractures Bone destruction Joint fragmentation and dislocation
Magnetic resonance imaging*	

Osteomyelitis	Charcot neuro-osteoarthropathy

Low focal signal intensity on T1-weighted images High focal signal on T2-weighted images High bone marrow signal in short tau inversion recovery (STIR) sequences Less specific or secondary changes: Cortical disruption Adjacent cutaneous ulcer Soft-tissue mass Sinus tract formation Adjacent soft-tissue inflammation or oedema	Altered bone marrow signal manifested by low signal intensity in the subchondral bone on both T1 and T2 weighted images Signal intensity abnormalities demonstrated by osteosclerosis and cystlike lesion Cortical fragmentation Joint deformity or subluxation Bone marrow oedema pattern: Tends to be periarticular and subchondral Predominant midfoot involvement Deformity is common along with bony debris Overlying skin is usually intact but may be oedematous

* For both modalities, bony changes are often accompanied by contiguous soft-tissue swelling.

Newer imaging techniques have been developed in the past few years. Fluorodeoxyglucose-positron emission tomography (FDG-PET) is a nuclear imaging technique that uses radiolabelled tracer 2-deoxy-2-[^18^F]fluoro-d-glucose (FDG), a marker of increased intracellular glucose metabolism. F^18^ FDG uptake is increased in both infection and CN (67). In one study, however, the mean standardised uptake value (SUV), a relative measure of FDG uptake, was 1.3±0.4 in CN compared with 4.38±1.39 in DFO (68). In cases having both CN and DFO, the SUV exceeded 6.5. In another study, the sensitivity and specificity of F^18^ FDG in CN were 100% and 93%, respectively, while the rates were 76.9% and 75%, respectively for MRI (68). Another new imaging technique is single-photon emission computed tomography/computed tomography (SPECT/CT), a fusion of scintigraphic and morphologic images. Reports have suggested that it is more accurate than planar scintigraphy alone, correctly differentiating DFO and contiguous soft-tissue infection in 97% of cases, compared with 59% for planar scintigraphy (69, 70). A recently reported study found that when ^67^Ga SPECT/CT imaging was combined with bedside percutaneous bone puncture the sensitivity and specificity were 88.0% and 93.6%, respectively, and the positive and negative predictive values were 91.7% and 90.7%, respectively (71).

In an effort to provide guidance for diagnosing osteomyelitis in the diabetic foot, an international panel of experts proposed consensus criteria with a novel approach that combines various findings to produce a probability, as shown in Table 4, (16). These criteria have not yet been tested to see if they are valid. As we have discussed, combining a variety of clinical, laboratory and imaging studies allows clinicians to differentiate DFO from CN in most cases. However, in cases where the diagnosis remains in doubt, or information on the causative pathogen is crucial, bone biopsy remains the diagnostic criterion standard for definitively diagnosing bone disorders in the foot of persons with diabetes.

**Table 4:** Proposed consensus criteria for diagnosing osteomyelitis in the diabetic foot (16)

Category	Criteria	Post-test probability of osteomyelitis	Management advice
Definite (beyond reasonable doubt)	Bone sample with positive culture AND positive histology OR Purulence in bone found at surgery OR A traumatically detached bone fragment removed from ulcer by podiatrist/surgeon OR Intraosseous abscess found on MRI OR Any two probable criteria OR one probable and two possible criteria OR, any four possible criteria below	*>*90%	Treat for osteomyelitis
Probable (more likely than not)	Visible cancellous bone in ulcer OR MRI showing bone oedema with other signs of osteomyelitis OR Bone sample with positive culture but negative or absent histology OR Bone sample with positive histology but negative or absent culture OR Any two possible criteria below	51–90%	Consider treating, but further investigation may be needed
Possible (but on balance, less rather than more likely)	Plain X-rays show cortical destruction OR MRI shows bone oedema OR cloaca, OR Probe-to-bone-positive OR, Visible cortical bone OR ESR *>*70mm/hour with no other plausible explanation OR Non-healing wound despite adequate offloading and perfusion for *>*6 weeks OR ulcer of *>*2 weeks duration with clinical evidence of infection	10–50%	Treatment may be justifiable, but further investigation usually advised
Unlikely	No signs or symptoms of inflammation AND normal X-rays AND ulcerpresent for *<*2 weeks or absent AND any ulcer present is superficial OR Normal MRI OR Normal bone scan	*<*10%	Usually no need for further investigation or treatment
